# Clinical validity of non-contrast-enhanced VI-RADS: prospective study using 3-T MRI with high-gradient magnetic field

**DOI:** 10.1007/s00330-022-08813-4

**Published:** 2022-05-12

**Authors:** Masanaka Watanabe, Satoru Taguchi, Haruhiko Machida, Mitsuhiro Tambo, Yuhei Takeshita, Toshiya Kariyasu, Keita Fukushima, Yuta Shimizu, Takatsugu Okegawa, Hiroshi Fukuhara, Kenichi Yokoyama

**Affiliations:** 1 Department of Radiology, Kyorin University School of Medicine, 6-20-2 Shinkawa, Mitaka, Tokyo, 181-8611 Japan; 2 Department of Urology, Kyorin University School of Medicine, 6-20-2 Shinkawa, Mitaka, Tokyo, 181-8611 Japan; 3 Department of Radiology, Tokyo Women’s Medical University Medical Center East, 2-1-10 Nishiogu, Arakawa, Tokyo, 116-8567 Japan

**Keywords:** Bladder cancer, Deep learning, Magnetic resonance imaging, Prospective, Vesical Imaging-Reporting and Data System (VI-RADS)

## Abstract

**Objectives:**

To develop a modified Vesical Imaging Reporting and Data System (VI-RADS) without dynamic contrast-enhanced imaging (DCEI), termed “non-contrast-enhanced VI-RADS (NCE-VI-RADS)”, and to assess the additive impact of denoising deep learning reconstruction (dDLR) on NCE-VI-RADS.

**Methods:**

From January 2019 through December 2020, 163 participants who underwent high-gradient 3-T MRI of the bladder were prospectively enrolled. In total, 108 participants with pathologically confirmed bladder cancer by transurethral resection were analyzed. Tumors were evaluated based on VI-RADS (scores 1–5) by two readers independently: an experienced radiologist (reader 1) and a senior radiology resident (reader 2). Conventional VI-RADS assessment included all three imaging types (T2-weighted imaging [T2WI], diffusion-weighted imaging [DWI], and dynamic contrast-enhanced imaging [DCEI]). Also evaluated were NCE-VI-RADS comprising only non-contrast-enhanced imaging types (T2WI and DWI), and “NCE-VI-RADS with dDLR” comprising T2WI processed with dDLR and DWI. All systems were assessed using receiver-operating characteristic curve analysis and simple and/or weighted *κ* statistics.

**Results:**

Muscle invasion was identified in 23/108 participants (21%). Area under the curve (AUC) values for diagnosing muscle invasion were as follows: conventional VI-RADS, 0.94 and 0.91; NCE-VI-RADS, 0.93 and 0.91; and “NCE-VI-RADS with dDLR”, 0.96 and 0.93, for readers 1 and 2, respectively. Simple *κ* statistics indicated substantial agreement for NCE-VI-RADS and almost perfect agreement for conventional VI-RADS and “NCE-VI-RADS with dDLR” between the two readers.

**Conclusion:**

NCE-VI-RADS achieved predictive accuracy for muscle invasion comparable to that of conventional VI-RADS. Additional use of dDLR improved the diagnostic accuracy of NCE-VI-RADS.

**Key Points:**

*• Non-contrast-enhanced Vesical Imaging Reporting and Data System (NCE-VI-RADS) was developed to avoid risk related to gadolinium-based contrast agent administration.*

*• NCE-VI-RADS had predictive accuracy for muscle invasion comparable to that of conventional VI-RADS.*

*• The additional use of denoising deep learning reconstruction (dDLR) might further improve the diagnostic accuracy of NCE-VI-RADS.*

**Supplementary Information:**

The online version contains supplementary material available at 10.1007/s00330-022-08813-4.

## Introduction

The Vesical Imaging Reporting and Data System (VI-RADS) is emerging as a standardized method for evaluating the magnetic resonance imaging (MRI) findings of bladder cancer (BC) that focuses on the diagnosis of muscle invasion [1]. Since VI-RADS was proposed in 2018, many studies [2–27] have validated its clinical utility and usefulness, including prospective studies [6, 7, 12, 20, 24, 26]. However, conventional VI-RADS has a potential limitation because it includes dynamic contrast-enhanced imaging (DCEI) as an indispensable component. The intravenous administration of gadolinium-based contrast agents has in rare cases caused allergy, nephrogenic systemic fibrosis, renal failure, and gadolinium deposition in the brain [28, 29]. Therefore, there is a clinical need to establish an alternative reporting system that does not use DCEI. Furthermore, MRI examination time and medical costs would be reduced if DCEI could be reasonably omitted from VI-RADS [24].

Denoising deep learning reconstruction (dDLR) has recently been applied in the clinical setting following the introduction of state-of-the-art 3-T MRI scanners with maximal gradient magnetic field of 100 mT/m [30]. These high-gradient (HG) MRI scanners can produce thinner slice images at the same bandwidth without additional scan time. The additional use of dDLR can retrospectively improve signal-to-noise ratio (SNR) in high-resolution MRI. dDLR comprises two steps: training and inference. In the training step, a deep learning model is built using large datasets, and in the inference step, noise is removed using the built model. The training step is performed before the model is installed into the MRI system, and the inference step is executed with the built model for datasets acquired with a clinical scanner [30]. It has been hypothesized that acquisition of non-contrast-enhanced high-resolution MRI with preserved SNR by combined use of HG MRI scanner and dDLR might compensate for a lack of DCEI in patients contraindicated for contrast agent administration. In this context, the present study aimed to develop a non-contrast-enhanced VI-RADS (NCE-VI-RADS), and to assess the additive impact of dDLR on NCE-VI-RADS in prospectively collected participants undergoing bladder MRI with the HG MRI scanner.

## Materials and methods

This prospective study was approved by the internal institutional review board of Kyorin University School of Medicine (approval No. 620), and written informed consent was obtained from each participant.

### Data collection and participant population

**Fig. 1:**
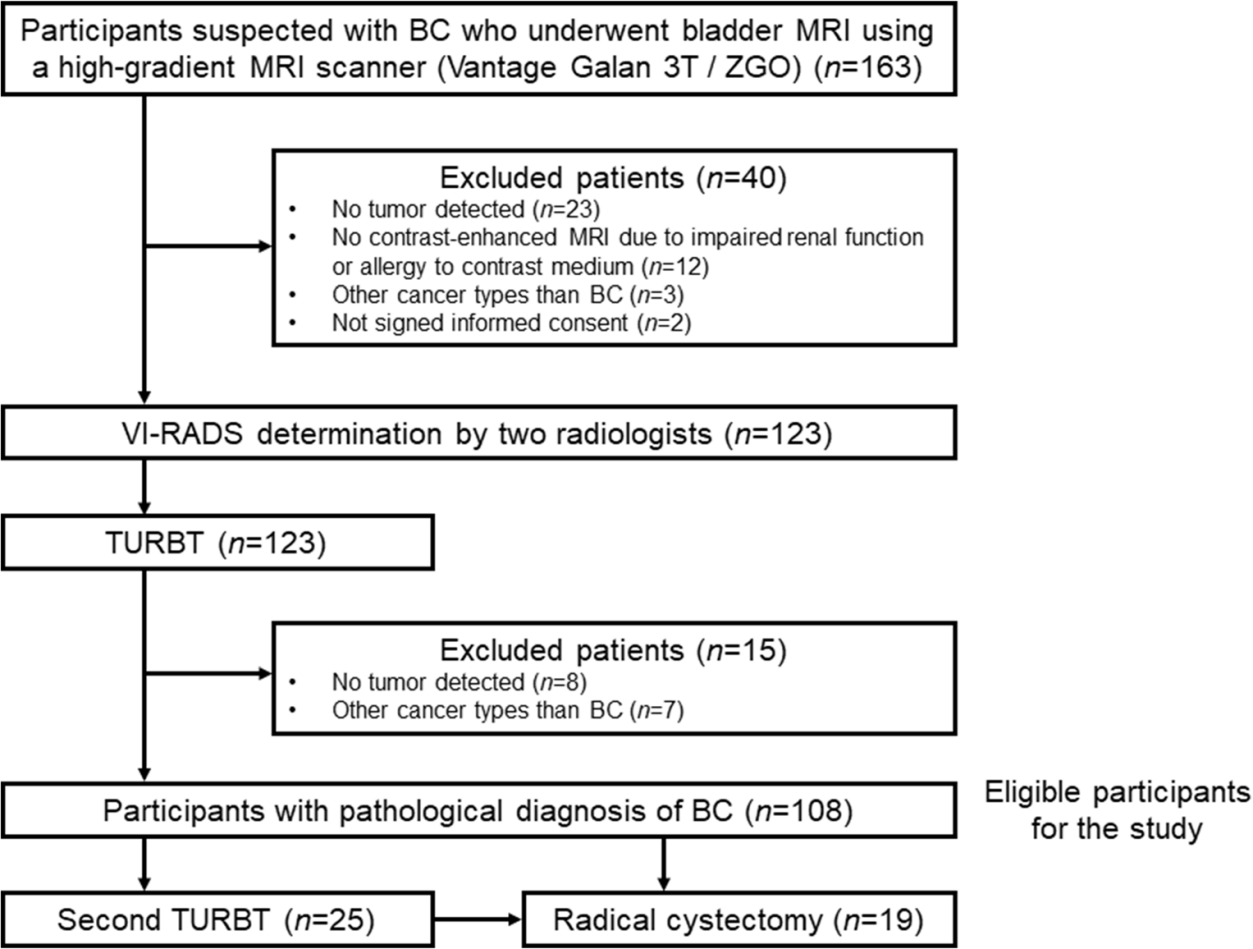
CONSORT diagram. BC, bladder cancer; TURBT, transurethral resection of bladder tumor; VI-RADS, Vesical Imaging Reporting and Data System

From January 2019 through December 2020, 163 consecutive participants undergoing bladder MRI using a 3-T HG scanner before transurethral resection of bladder tumor (TURBT) at a single institution were prospectively enrolled. The exclusion criteria were age < 20 years, contraindications to MRI, and participant refusal. Based on the pathological findings of the initial TURBT, 108 participants who confirmed urothelial BC pathologically were included in this analysis (Fig. 1). The cohort included 68 participants who were previously reported [20]. The most recent follow-up information was obtained in March 2021.

### Multiparametric MRI examination

All multiparametric MRI examinations were performed using the same HG MRI scanner (Vantage Galan 3T/ZGO, Canon Medical Systems) without administration of antispasmodic agent. This scanner incorporates dDLR technology (Advanced intelligent Clear-IQ Engine [AiCE], Canon Medical Systems) that has been developed using deep learning to improve SNR in high-resolution imaging [30].

The scan protocol included 2- and 4-mm-slice axial and oblique T2-weighted imaging (T2WI), 1.5- and 4-mm-slice axial diffusion-weighted imaging (DWI), and 1-mm-slice oblique DCEI. In addition, 1.5-mm-slice multiplanar reformation coronal and oblique DWI were reconstructed from the axial images [20]. Supplementary Table **1** lists the parameter settings in detail. If bladder distension was insufficient, the participant was asked to drink 300 mL of water prior to the acquisition of axial and oblique T2WI, axial DWI, and oblique DCEI. The lesion presumed to be the most invasive was selected as the index lesion. The optimal oblique plane for assessing the VI-RADS score was determined as perpendicular to the base of that lesion. Gadolinium-based contrast agent (gadoteridol; ProHance, Bracco-Eisai) was administered by power injector (Sonic Shot 7; Nemoto Kyorindo) via the right antecubital vein using a 22-gauge plastic intravenous catheter, at a dose of 0.2 mmol/kg of body weight and flow rate of 2 mL/s. DCEI was then acquired at 30, 60, 90, and 120 s.

### Image evaluation by VI-RADS and NCE-VI-RADS

All MRI images were evaluated based on VI-RADS [1] by two independent readers without knowledge of the surgical or histologic findings: reader 1 (M.W., a board-certified radiologist with 7 years of experience in urogenital radiology) and reader 2 (Y.T., a senior radiology resident with 1 year of experience in urogenital radiology). The imaging findings were scored as 1–5 for each of the T2WI, DWI, and DCEI categories. These were assessed separately prior to determination of the VI-RADS and NCE-VI-RADS scores. The conventional VI-RADS score (1–5) utilized the results of all three imaging categories (T2WI, DWI, and DCEI), whereas the NCE-VI-RADS score (1–5) utilized only the results of the NCE imaging categories (T2WI and DWI). In conventional VI-RADS and NCE-VI-RADS, the T2WI category was assessed on both 2- and 4-mm-slice T2WI without dDLR. However, because it is generally recommended that 4-mm-slices should be used for assessment, we mainly assessed these 4-mm images in the present study. We adopted a cutoff value of ≥ 4 for conventional VI-RADS because of its high specificity for the diagnosis of muscle invasion [31]. Negative NCE-VI-RADS was defined as a score of < 4 for both the T2WI and DWI categories, and positive NCE-VI-RADS was defined as a score of ≥ 4 for either of the T2WI or DWI categories (or both). As an exploratory analysis, “NCE-VI-RADS with dDLR” assessed on 2-mm-slice T2WI processed with dDLR (T2WI+dDLR) and DWI were defined similarly. Therefore, “NCE-VI-RADS with dDLR” represents a modified version of NCE-VI-RADS in which T2WI was replaced by T2WI+dDLR.

### TURBT and pathological diagnosis

After bladder MRI, each participant underwent conventional monopolar or bipolar TURBT with random bladder biopsy. Experienced board-certified pathologists who were blinded to the MRI results reviewed all specimens to assess the histological type, grade, and stage of the tumors according to the 2004/2016 World Health Organization grading systems [32] and the 2017 American Joint Committee on Cancer/Union for International Cancer Control TNM staging system [33]. A second TURBT was performed when indicated, according to the current clinical guidelines [34]. The presence or absence of muscle invasion of BC by TURBT was considered the definitive diagnosis.

### Statistical analysis

The predictive accuracies of muscle invasion of BC based on the T2WI, DWI, and DCEI categories, conventional VI-RADS, NCE-VI-RADS, and “NCE-VI-RADS with dDLR” were assessed using the receiver-operating characteristic (ROC) curve analysis as ordinal (1–5) and/or nominal (< 4 vs. ≥ 4) scales. Sensitivity, specificity, positive predictive value, negative predictive value, accuracy, and area under the curve (AUC) were calculated using a 2 × 2 contingency table for different cut-points of the VI-RADS score. Simple and/or weighted *κ* statistics were used to estimate inter-reader agreement. The AUC values were compared between conventional VI-RADS vs. NCE-VI-RADS with or without dDLR within each reader. All statistical analyses were performed using commercially available statistical software (JMP Pro version 15.0.0; SAS Institute). *p* < 0.05 was considered statistically significant.

## Results

A total of 108 participants, 88 (81%) men and 20 (19%) women, were included in this validation study. The median participant age was 74.0 years (interquartile range [IQR], 67.0–81.0 years). Detailed characteristics of the 108 participants are shown in Table 1. Muscle invasion of BC was identified by the initial TURBT in 23/108 participants (21%). After the initial TURBT, 25/108 (23%) participants underwent a second TURBT; however, no participant was upstaged to ≥ T2. The pathological findings identified by the second TURBT were as follows: no residual disease (*n* = 13), atypical urothelium (*n* = 2), papillary urothelial neoplasm of low malignant potential (*n* = 1), Ta (*n* = 1), Tis (*n* = 5), and T1 (*n* = 3). After TURBT, a total of 19 participants underwent radical cystectomy and the obtained definitive diagnoses were as follows: pT0 (*n* = 2), pTis (*n* = 3), pT1 (*n* = 1), pT1+pTis (*n* = 1), pT2 (*n* = 2), pT2+pTis (*n* = 1), pT3a (*n* = 3), pT3b (*n* = 4), pT4a (*n* = 1), and unknown due to surgery at another institution (*n* = 1).

**Table 1:** Detailed participant characteristics (*n* = 108)

Parameter	Value
Median age (interquartile range), years	74 (67–81)
Gender, no. (%):	
Male	88 (81)
Female	20 (19)
Previous history of urothelial cancers, no. (%):	
None	95 (88)
Non-muscle-invasive bladder cancer	6 (6)
Upper tract urothelial cancer	4 (4)
Both	3 (3)
No. of lesions (%):	
Unifocal	54 (50)
Multifocal	54 (50)
Tumor size, no. (%):	
< 30 mm	82 (76)
≥ 30 mm	26 (24)
Pathological T stage, no. (%):	
Ta	48 (44)
Tis	3 (3)
T1	34 (31)
≥ T2	23 (21)
Concomitant carcinoma in situ, no. (%)	28 (26)
Pathological grade, no. (%):	
Low grade	26 (24)
High grade	82 (76)
Histological type, no. (%):	
Pure urothelial carcinoma	94 (87)
Urothelial carcinoma with squamous differentiation	9 (8)
Urothelial carcinoma with glandular differentiation	3 (3)
Urothelial carcinoma with squamous and glandular differentiations	1 (1)
Urothelial carcinoma with sarcomatoid differentiation	1 (1)

**Fig. 2:**
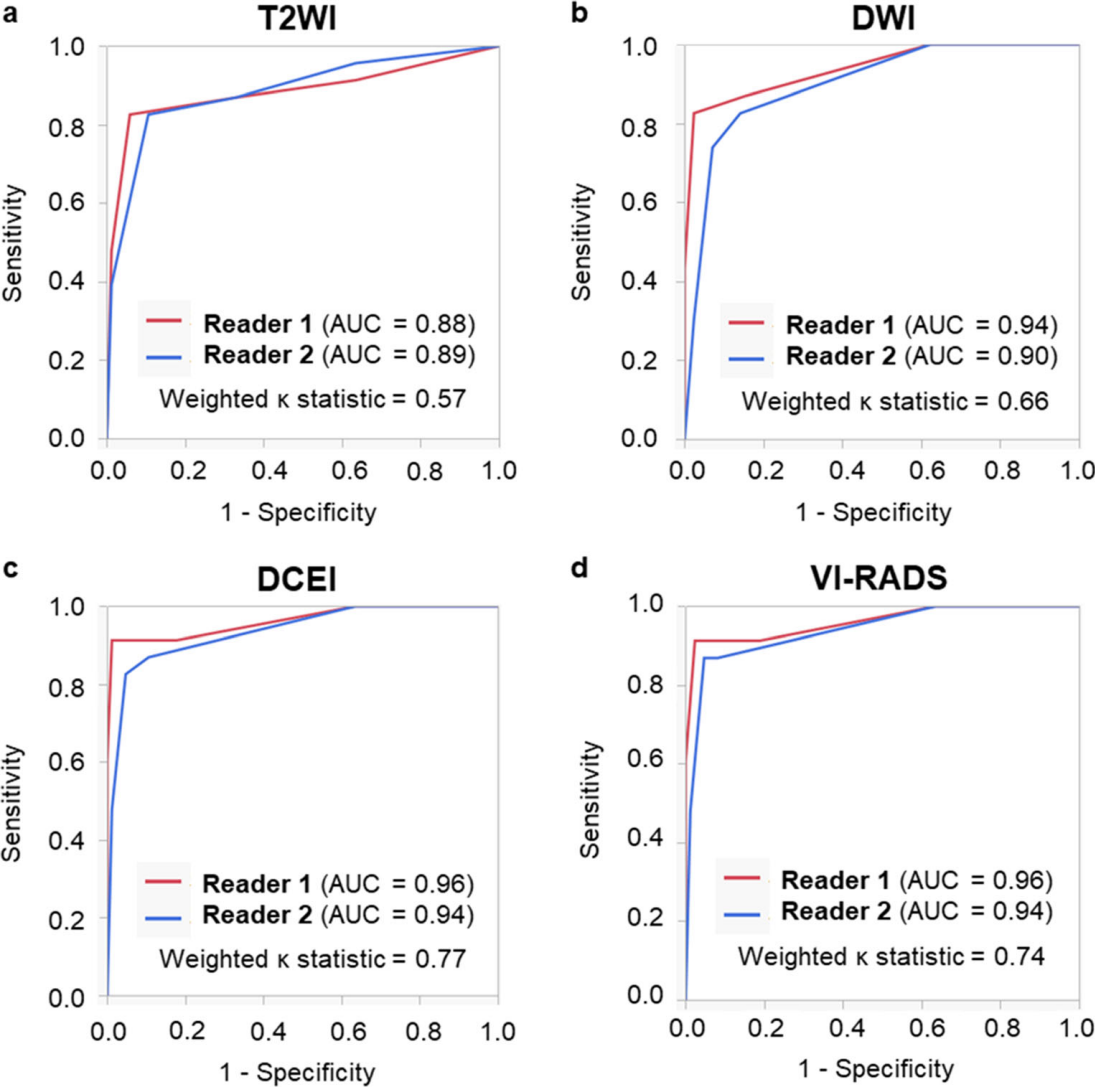
ROC curve analyses of categories by (**a**) T2WI, (**b**) DWI, (**c**) DCEI, and (**d**) conventional VI-RADS score for muscle invasion by the two readers. AUC, area under the curve; DCEI, dynamic contrast-enhanced imaging; DWI, diffusion-weighted imaging; ROC, receiver operating characteristic; T2WI, T2-weighted imaging; VI-RADS, Vesical Imaging Reporting and Data System.

Figure 2 shows the ROC curve analyses for each of the two readers of the T2WI, DWI, and DCEI categories, and the conventional VI-RADS score for diagnosing muscle invasion. The AUC values for T2WI, DWI, and DCEI categories, and for conventional VI-RADS score were 0.88–0.89, 0.90–0.94, 0.94–0.96, and 0.94–0.96, respectively. Weighted *κ*statistics indicated moderate agreement (0.41–0.60) for T2WI category and substantial agreement (0.61–0.80) for the other parameters between the two readers.

**Fig. 3:**
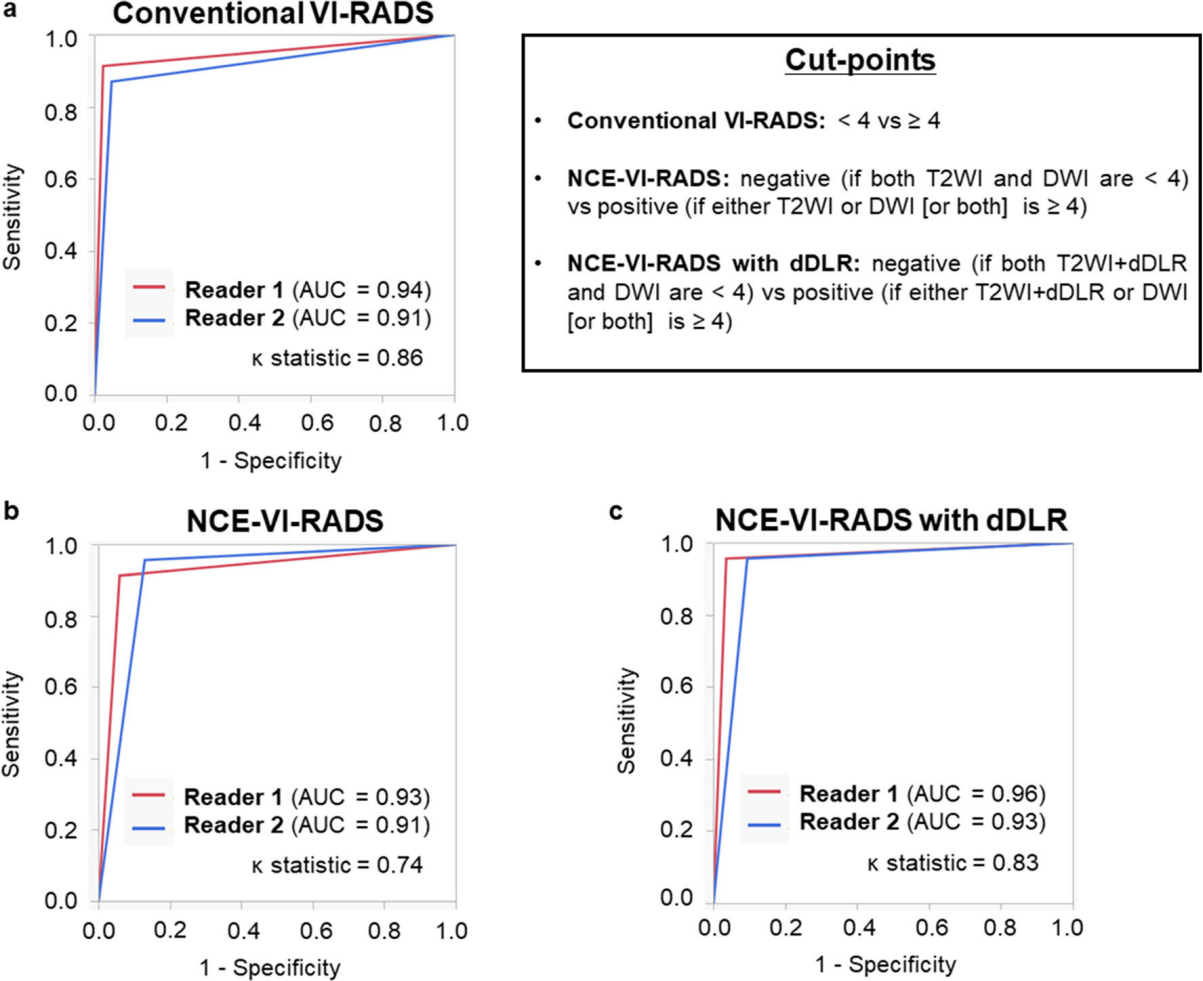
ROC curve analyses of (**a**) conventional VI-RADS, (**b**) NCE-VI-RADS, and (**c**) “NCE-VI-RADS with dDLR” for diagnosing muscle invasion by the two readers. As NCE-VI-RADS and “NCE-VI-RADS with dDLR” were defined as categorical variables (negative vs positive), conventional VI-RADS was presented in the categorical manner (< 4 vs ≥ 4). AUC, area under the curve; dDLR, denoising deep learning reconstruction; NCE-VI-RADS, non-contrast-enhanced Vesical Imaging Reporting and Data System; ROC, receiver operating characteristic; VI-RADS, Vesical Imaging Reporting and Data System

Figure 3 shows the ROC curve analyses of conventional VI-RADS, NCE-VI-RADS, and “NCE-VI-RADS with dDLR” for diagnosing muscle invasion, for each of the two readers. Because NCE-VI-RADS and “NCE-VI-RADS with dDLR” were defined as categorical variables (negative vs. positive), conventional VI-RADS was also presented in this manner (< 4 vs. ≥ 4). The AUC values for conventional VI-RADS, NCE-VI-RADS, and “NCE-VI-RADS with dDLR” were 0.91–0.94, 0.91–0.93, and 0.93–0.96, respectively. Simple *κ* statistics indicated substantial agreement (0.61–0.80) for NCE-VI-RADS and almost perfect agreement (0.81–1.00) for conventional VI-RADS and “NCE-VI-RADS with dDLR” between the two readers. The AUC values were also compared between conventional VI-RADS vs. NCE-VI-RADS and conventional VI-RADS vs. “NCE-VI-RADS with dDLR” within each reader, none of which indicated a significant difference (reader 1: *p* = 0.08 and *p* = 0.48; reader 2: *p* = 0.95 and *p* = 0.54).

Supplementary Table 2 lists the accuracy of diagnosing muscle invasion based on each parameter (T2WI, T2WI+dDLR, DWI, DCEI, conventional VI-RADS, NCE-VI-RADS, and “NCE-VI-RADS with dDLR”) with a cut-point of ≥ 4 for each reader. Simple *κ* statistics between the two readers showed either substantial (0.61–0.80) or almost perfect (0.80–1.00) agreement for all parameters.

**Fig. 4:**
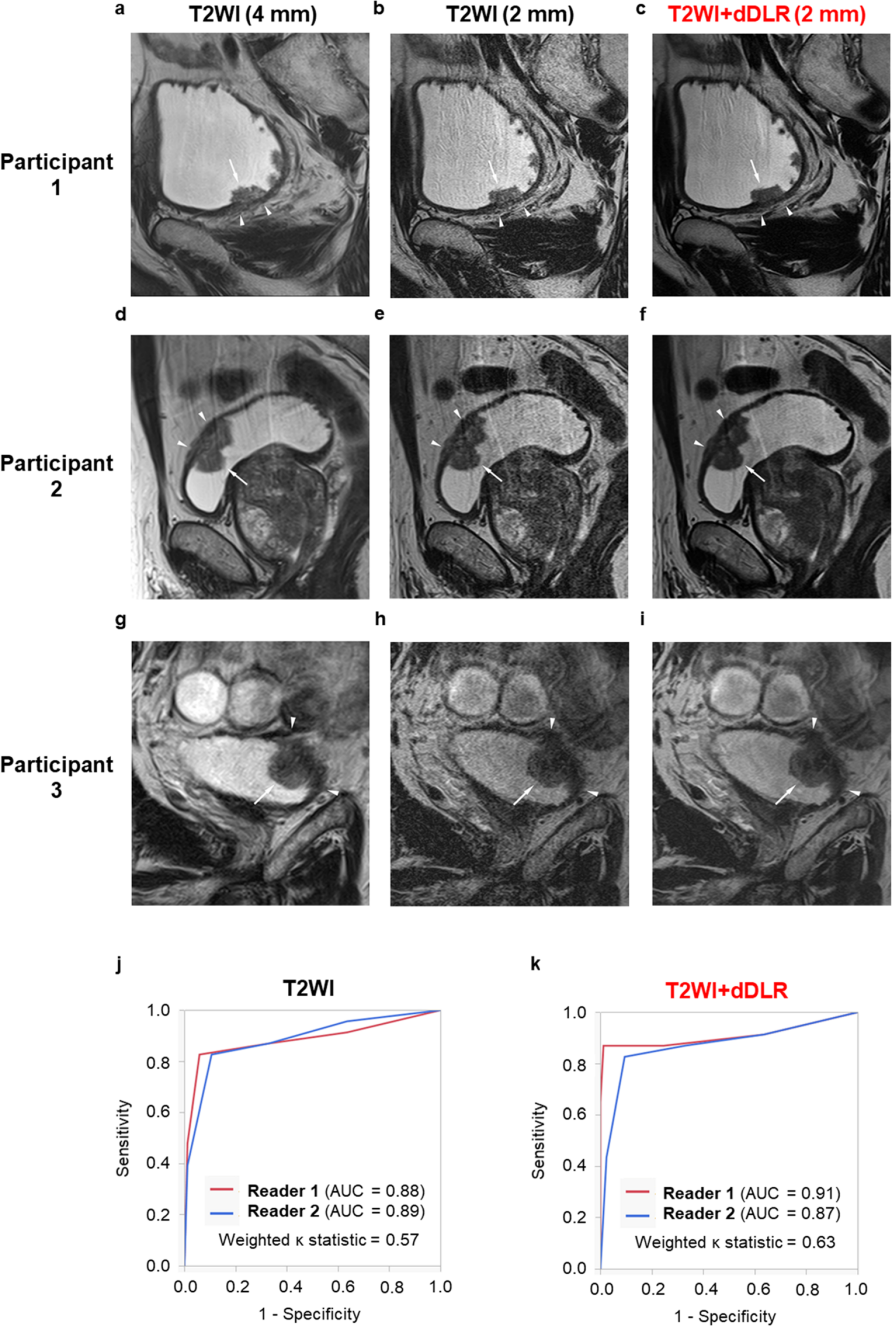
Representative MRI images show the usefulness of combining dDLR with T2WI in three cases. The first two cases (participants 1 and 2) were pathologically negative for muscle invasion. On 4-mm-slice oblique T2WI (**a**, **d**), moderate signal representing the tumor (arrow) appears to disrupt low signal representing the muscularis propria (arrowheads), suggesting VI-RADS category 4 or 5. On 2-mm-slice oblique T2WI (**b**, **e**), low signal representing the muscularis propria (arrowheads) is obscure due to low SNR, also suggesting VI-RADS category 4 or 5. In contrast, on 2-mm-slice oblique T2WI with dDLR (**c**, **f**), low signal representing the muscularis propria (arrowheads) appears to be continuous, which correctly indicates VI-RADS category 3 or lower. The last case (participant 3) was pathologically positive for muscle invasion. On 4-mm-slice oblique T2WI (**g**) and 2-mm-slice oblique T2WI (**h**), the border between low signal representing the muscularis propria (arrowheads) and moderate signal representing the tumor (arrow) appears to be indistinct but almost intact, with no definite disruption of the low signal by moderate signal, suggesting VI-RADS category 3. In contrast, on 2-mm-slice oblique T2WI with dDLR (**i**), moderate signal representing the tumor (arrow) appears to disrupt low signal representing the muscularis propria (arrowheads), which correctly indicates VI-RADS category 4 or 5, and is visible due to higher spatial resolution and SNR. The ROC curve analyses of categories by T2WI alone (**j**) and T2WI+dDLR (**k**) are shown below the images. AUC, area under the curve; dDLR, denoising deep learning reconstruction; MRI, magnetic resonance imaging; ROC, receiver operating characteristic; SNR, signal-to-noise ratio; T2WI, T2-weighted imaging.

Representative images showing the usefulness of T2WI+dDLR in three participants are shown in Fig. 4. The ROC curve analyses of the T2WI alone and T2WI+dDLR categories are shown below the images. Although there was no significant difference, the AUC increased from 0.88 with T2WI alone to 0.91 with T2WI+dDLR for reader 1 (*p* = 0.35), but remained almost unchanged from 0.89 to 0.87 for reader 2 (*p* = 0.42). Weighted *κ* statistics between the two readers increased from 0.57 with T2WI alone to 0.63 with T2WI+dDLR.

## Discussion

In the present study, the predictive accuracy of muscle invasion of NCE-VI-RADS comprising only T2WI and DWI was examined. NCE-VI-RADS was defined as negative when both T2WI and DWI were < 4, and as positive when either T2WI or DWI (or both) was ≥ 4. The predictive accuracy of NCE-VI-RADS was assessed in comparison with conventional VI-RADS for muscle invasion in a prospective cohort of 108 participants with BC, using images acquired by a 3-T HG MRI scanner. The AUC for NCE-VI-RADS was comparable to that for conventional VI-RADS for each reader (reader 1: 0.93 vs. 0.94, *p* = 0.08; reader 2: 0.91 vs. 0.91, *p* = 0.95), which might suggest the equivalence (or non-inferiority) of NCE-VI-RADS to conventional VI-RADS. Furthermore, the additive impact of dDLR on NCE-VI-RADS was assessed. Although the difference was not significant, AUC was higher for NCE-VI-RADS+dDLR than for conventional VI-RADS for each reader (reader 1: 0.96 vs. 0.94, *p* = 0.48; reader 2: 0.93 vs. 0.91, *p* = 0.54). These preliminary data suggest that the additional use of dDLR could possibly further improve the diagnostic accuracy of NCE-VI-RADS.

To the best of our knowledge, only one previous study has assessed the utility of VI-RADS in a “contrast-agent-free” setting [24]. Delli Pizzi et al compared the results of non-contrast biparametric MRI including T2WI and DWI with those of standard multiparametric MRI comprising T2WI, DWI, and DCEI using a prospective cohort of 38 participants and reported comparable diagnostic accuracy between the biparametric and multiparametric protocols for the detection of muscle-invasive BC [24]. The present prospective study enrolled three times as many participants as that study, and obtained similar results. In addition, five previous prospective studies [6, 7, 12, 20, 26] have aimed to validate the utility of conventional VI-RADS. Makboul et al prospectively enrolled 50 patients and reported AUC of 0.83 for conventional VI-RADS [6]. Del Giudice et al prospectively enrolled 231 patients and assessed the ability of conventional VI-RADS (< 3 vs. ≥ 3) to discriminate between non-muscle-invasive and muscle-invasive BC. They concluded that a conventional VI-RADS score of ≥ 3 could be a predictor of understaged muscle-invasive BC after initial TURBT, leading to improved selection of candidates for a second TURBT [7]. Marchioni et al prospectively enrolled 38 patients with a total of 68 BC lesions and reported that a conventional VI-RADS score of ≥ 4 achieved the highest sensitivity (85.7%) and specificity (86.9%) among the different cut-points [12]. Taguchi et al prospectively enrolled 68 patients who underwent 3-T HG MRI scanner, and demonstrated that the accuracy of diagnosing muscle invasion by conventional VI-RADS of ≥ 4 was 94% (AUC = 0.92). They also showed the potential utility of T2WI+dDLR in an exploratory analysis [20]. Metwally et al have recently reported a prospective multicenter study of 331 patients. They reported that the optimal cut-point for predicting muscle invasion was a VI-RADS score of > 3 (AUC = 0.94) after the first TURBT and VI-RADS of > 2 (AUC = 0.96) after the second TURBT [26]. The utility of conventional VI-RADS has been increasingly confirmed by cumulative evidence. The present study utilized HG MRI scanner, which enables slice thickness to be decreased without additional scan time, to acquire 2-mm-slice T2WI. Slice thickness of 3–4 mm is generally recommended to maximize spatial resolution while maintaining SNR in T2WI [1]. HG MRI scanner applies dDLR to improve SNR and achieve high-resolution images while preserving SNR, and is in use in the clinical setting [30, 35]. The results of a previous study [20] and the present findings suggest that the additional use of dDLR might enable a more accurate prediction of muscle invasion in patients with BC. It also would be beneficial in patients who are contraindicated for contrast agent administration and reduce the length of the examination and avoid the cost of contrast agent.

The major limitations of the present study are its single-institutional design and relatively small sample size. In addition, the HG MRI scanner used in the present study is not yet widely available. Further prospective multi-institutional studies with larger populations are warranted to confirm our results.

In conclusion, in this prospective study employing a 3-T HG MRI scanner, NCE-VI-RADS achieved comparable predictive accuracy for muscle invasion in bladder cancer to that of conventional VI-RADS. The additional use of dDLR might further improve the diagnostic accuracy of NCE-VI-RADS.

## Supplementary Information

ESM 1 (DOCX 21 kb)

## Abbreviations and acronyms

AUC: Area under the curve
BC: Bladder cancer
DCEI: Dynamic contrast-enhanced imaging
dDLR: Denoising deep learning reconstruction
DWI: Diffusion-weighted imaging
HG: High-gradient
MRI: Magnetic resonance imaging
NCE-VI-RADS: Non-contrast-enhanced Vesical Imaging Reporting and Data System
ROC: Receiver operating characteristic
SNR: Signal-to-noise ratio
T2WI: T2-weighted imaging
VI-RADS: Vesical Imaging Reporting and Data System

## Declarations

### Guarantor

The scientific guarantor of this publication is Prof. Kenichi Yokoyama (Department of Radiology, Kyorin University School of Medicine).

### Statistics and biometry

No complex statistical methods were necessary for this paper.

### Study subjects or cohorts overlap

Some study subjects have been previously reported in the following study:

Taguchi, S., et al. Prospective Validation of Vesical Imaging-Reporting and Data System Using a Next-Generation Magnetic Resonance Imaging Scanner-Is Denoising Deep Learning Reconstruction Useful? J Urol. 2021 205:686–692.

### Methodology

• prospective

• diagnostic or prognostic study

• performed at one institution
