# Supplementary material for: Clinical validity of non-contrast-enhanced VI-RADS: prospective study using 3-T MRI with high-gradient magnetic field
Source: Eur Radiol. 2022 May 12;32(11):7513–21. doi: 10.1007/s00330-022-08813-4 (PMC9668777; doi:10.1007/s00330-022-08813-4)
Supplement: Supplementary file 1 — (DOCX 21 kb) [file 330_2022_8813_MOESM1_ESM.docx]

**Supplementary Table 1** Detailed parameter settings for bladder MRI

|  | **T2WI (4 mm)** | **T2WI (2 mm)** | **DWI (4 mm)** | **DWI (1.5 mm)** | **DCEI** |
| --- | --- | --- | --- | --- | --- |
| **TR (ms)** | 5706 | 5792 | 5300 | 5300 | 3.8 |
| **TE (ms)** | 140 | 136 | 40 | 40 | 1.3 |
| **FOV (cm)** | 25 × 25 | 25 × 25 | 25 × 25 | 25 × 25 | 25 × 25 |
| **Matrix size** | 256 × 320 | 384 × 432 | 144 × 144 | 144 × 144 | 208 × 240 |
| **Slice thickness (mm)** | 4 | 2 | 4 | 1.5 | 1 |
| **Slice gap (mm)** | 0.5 | 0.2 | 0.5 | 0 | 0 |
| **FA (deg)** | 90 | 120 | 90 | 90 | 12 |
| **NAQ** | 2 | 1 | 1 | 1 | 1 |
| **AT (min:s)** | 1:49 | 2:37 | 1:36 | 3:12 | 0:26 |
| ***B* value (s/mm^2^)** | n.a. | n.a. | 0, 1000 | 0, 1000 | n.a. |

AT = acquisition time, DCEI = dynamic contrast-enhanced imaging, DWI = diffusion-weighted imaging, FA = flip angle, FOV = field of view, MRI = magnetic resonance imaging, NAQ = Number of acquisition time, TE = echo time, TR = reception time, T2WI = T2-weighted imaging

**Supplementary Table 2** Accuracy for diagnosing muscle invasion according to each parameter (T2WI, T2WI+dDLR, DWI, DCEI, the conventional VI-RADS, NCE-VI-RADS, or NCE-VI-RADS with dDLR) with cut-point of ≥ 4 and each reader (Reader 1 or 2)

| **Parameter** | **T2WI ≥ 4** | | **T2WI+dDLR ≥ 4** | | **DWI ≥ 4** | | **DCEI ≥ 4** | | **Conventional**  **VI-RADS ≥ 4** | | **NCE-VI-RADS ≥ 4** | | **NCE-VI-RADS with dDLR ≥ 4** | | |
| --- | --- | --- | --- | --- | --- | --- | --- | --- | --- | --- | --- | --- | --- | --- | --- |
|  | **Reader 1** | **Reader 2** | **Reader 1** | **Reader 2** | **Reader 1** | **Reader 2** | **Reader 1** | **Reader 2** | **Reader 1** | **Reader 2** | **Reader 1** | **Reader 2** | **Reader 1** | **Reader 2** |  |
| Sensitivity | 83% | 83% | 87% | 83% | 83% | 74% | 91% | 83% | 91% | 87% | 91% | 96% | 96% | 96% |  |
| Specificity | 94% | 89% | 99% | 91% | 98% | 94% | 99% | 95% | 98% | 95% | 94% | 87% | 96% | 91% |  |
| PPV | 79% | 68% | 95% | 70% | 90% | 77% | 95% | 83% | 91% | 83% | 81% | 67% | 88% | 73% |  |
| NPV | 95% | 95% | 97% | 95% | 95% | 93% | 98% | 95% | 98% | 96% | 98% | 99% | 99% | 99% |  |
| Accuracy | 92% | 88% | 96% | 89% | 94% | 90% | 97% | 93% | 96% | 94% | 94% | 89% | 96% | 92% |  |
| AUC | 0.88 | 0.86 | 0.93 | 0.87 | 0.90 | 0.84 | 0.95 | 0.89 | 0.94 | 0.91 | 0.93 | 0.91 | 0.96 | 0.93 |  |
| κ statistics | 0.75 | – | 0.79 | – | 0.74 | – | 0.86 | – | 0.86 | – | 0.74 | – | 0.83 | – |  |

AUC = area under the curve, DCEI = dynamic contrast-enhanced imaging, dDLR = denoising deep learning reconstruction, DWI = diffusion-weighted imaging, NCE-VI-RADS = non-contrast-enhanced Vesical Imaging Reporting and Data System, NPV = negative predictive value, PPV = positive predictive value, T2WI = T2-weighted imaging
